# Retraction: Digital inclusive finance, agricultural green technology innovation and agricultural carbon emissions: Impact mechanism and empirical test

**DOI:** 10.1371/journal.pone.0325328

**Published:** 2025-05-27

**Authors:** The PLOS One Editors

The *PLOS One* Editors retract this article [1] due to concerns about potential manipulation of the publication process. These concerns call into question the validity and provenance of the reported results. We regret that the issues were not identified prior to the article’s publication.

In addition, this article cites retracted work in References 17 and 31.

The author either did not respond directly or could not be reached.
